# Quantum Evaluation of a Comprehensive Set of Carboxylic Acid Bioisosteres: Gas versus Solvated Phases

**DOI:** 10.1021/acsomega.4c11714

**Published:** 2025-04-25

**Authors:** Alaa MA Osman, Alya A. Arabi

**Affiliations:** College of Medicine and Health Sciences, Department of Biochemistry and Molecular Biology, United Arab Emirates University, P.O. Box: AlAin 15551, United Arab Emirates

## Abstract

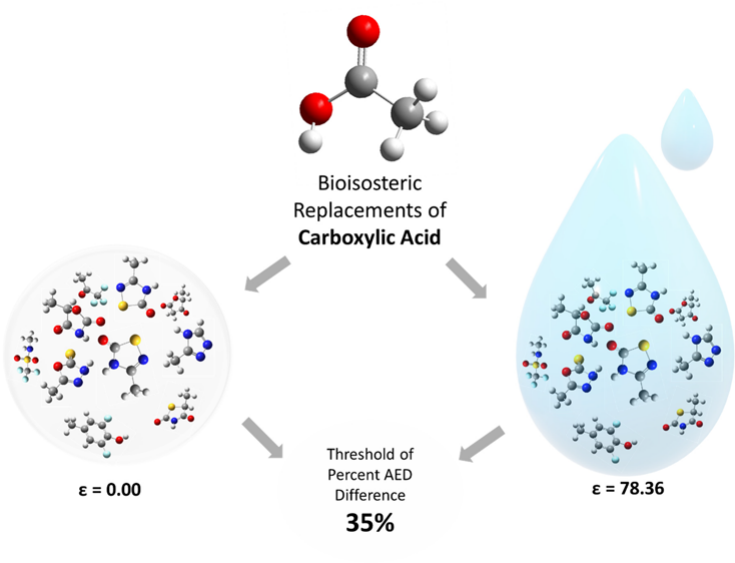

In drug design, bioisosterism is used to enhance the pharmacokinetic and pharmacodynamic properties of a drug molecule while maintaining its biological activity. This study investigates the use of a quantum tool, namely, the average electron density (AED) tool, in evaluating 54 experimentally tested nonclassical carboxylic acid bioisosteres. In total, 65 bioisosteres were considered, including both R and S enantiomers for 11 chiral moieties. The AED values of all bioisosteric moieties deviate by up to 32% compared to the carboxylic acid group, regardless of the medium, whether in the gas phase or implicitly solvated with water. This suggests that a 32% deviation threshold is reasonable for classifying potential carboxylic acid bioisosteres.

## Introduction

In drug design, bioisosterism involves enhancing the pharmacokinetic and pharmacodynamic properties of a lead compound by substituting a moiety with another, known as a bioisostere, while maintaining the intended biological activity or therapeutic effect. Bioisosteric replacements have been reported to improve many properties,^1−4^ including potency and effectiveness,^5^ absorption, distribution, metabolism, and excretion (ADME) properties,^6^ solubility, hydrophilicity, hydrophobicity,^6,7^ toxicological profile,^8^ resistance to metabolism resulting in an extended half-life,^9^ selectivity for more targeted effects,^10^ and physicochemical properties that enable alternative route of administration.^11^ Several methods have been reported to evaluate bioisosterism. Electrostatic potential (ESP) maps have been used to qualitatively explain the key-and-lock complementarity between a ligand and its receptor.^12−14^ However, ESP maps have been shown to be ambiguous in certain cases, potentially leading to inaccurate analyses or inconclusive results in the context of bioisosterism.^15−18^ Alternatively, other approaches include structure-based approaches (which depend on the structure of the receptor), such as the KRIPO tool based on pharmacophore fingerprints,^19^ and ligand-based approaches (which depend on the structure of the ligand), such as structure property relationship (SPR) methods^20,21^ and the qualitative AED tool.^15−17,22,23^

The AED tool was introduced in 2010 it was first tested on the carboxylate and tetrazole anion bioisosteric pair.^24^ Subsequent studies have tested the applicability of the AED tool on several nonclassical carboxylic acid bioisosteres, including methylsquarate,^17^ sulfonamides,^15,16^ isoxazole, tetrazol-5-one, oxadiazole, oxazolidinedione, and thiazolidinedione,^23^ and furan.^16^ These bioisosteric moieties were studied under various conditions, such as different protonation states (neutral and anionic molecules), different capping groups spanning a wide range of electronegativities (a hydrogen, chloro, methyl, benzyl, or amine group), different isodensity values that define atomic basins (0.0004, 0.001, and 0.002 au), and different surrounding milieus (molecules in vacuum *vs* molecules docked into their target proteins). One of the aims of this study is to evaluate the applicability of the AED tool on an expanded set of more than 50 experimentally tested nonclassical carboxylic acid bioisosteres. This study also explores the effect of R and S enantiomerism on the AED values. Additionally, this study aims to validate the AED tool for assessing similarities among bioisosteric moieties in drug molecules that are implicitly solvated with water. This represents a significant advancement, as previous validations were limited to molecules in the gas phase.^18,22,25,26^

## Computational Methods

**Figure 1:**
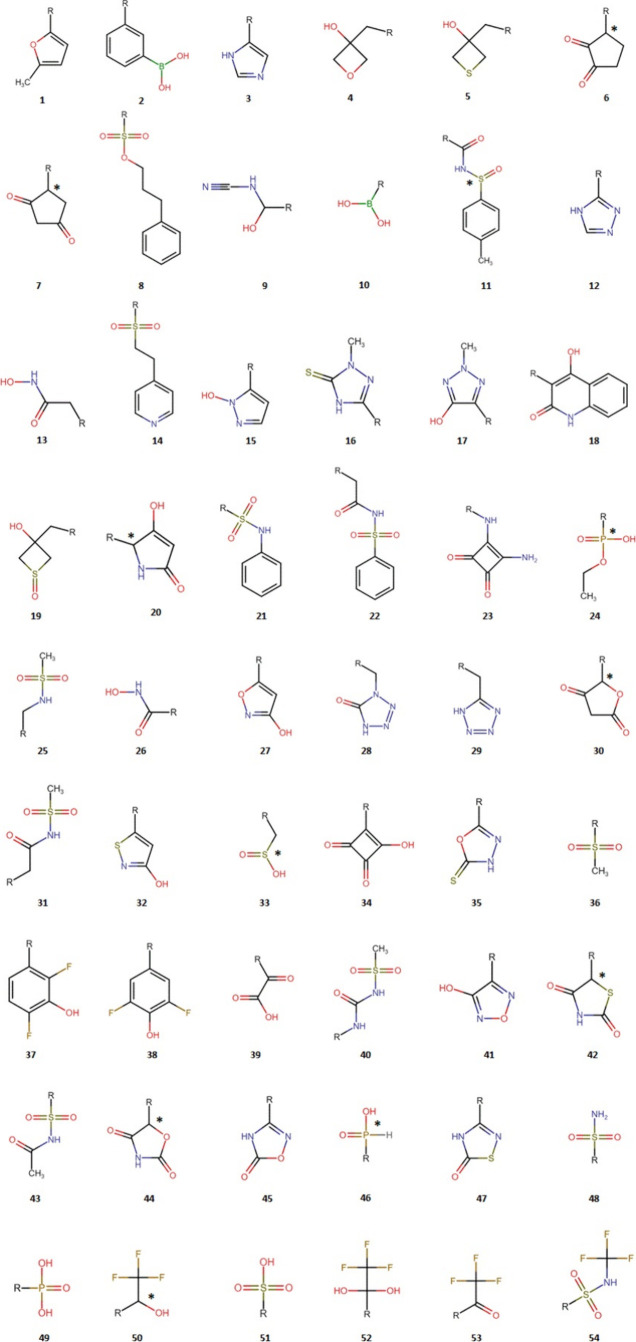
2D structures of the 54 carboxylic acid bioisosteres considered in this study, each capped with a methyl group (denoted by an R). Chiral centers are indicated by an asterisk.

A total of 54 nonclassical carboxylic acid bioisosteres were gathered from published experimental studies (see Table 1). Both R and S enantiomers of the chiral moieties (in molecules 6, 7, 11, 20, 24, 30, 33, 42, 44, 46, and 50) were considered, with the chiral centers denoted by an asterisk in Figure 1. Using GaussView 6.0,^27^ the bioisosteric moieties were built and capped with a methyl group. Using the Gaussian 16 package,^28^ all molecules were optimized, in vacuum and under implicit water solvation, using the integral equation formalism polarizable continuum model (IEFPCM)^29^ with a dielectric constant of 78.36^30^ to mimic water effects. The B3LYP/6–311++G(d,p)//B3LYP/6–311++G(d,p) level of theory was used with ″Tight″ self-consistent field (SCF) optimization and ultrafine pruned (99,590) grids. A vibrational frequency analysis was performed to confirm that the optimized geometries did not correspond to transition states. AIMALL^31^ was used to obtain the atomic properties (*i.e.*, volumes and the electron populations). The AED of each bioisosteric moiety was calculated using the following equation:
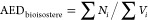
where ∑*N_i_* is the sum of the atomic electron populations, ∑*V_i_* is the sum of the atomic volumes, and *i* represents each atom in the bioisosteric moiety.

**Table 1:** List of the 54 Nonclassical Carboxylic Acid Bioisosteric Moieties Considered in This Study, Numbered as in Figure 1, along with Their Corresponding Literature Reference(s) and Their Reported Medicinal Application(s)^a^

**molecule**	**reference(s)**	**medicinal application(s)**
1	(32)	-EP1 receptor antagonist
2	(33−36)	-neuraminidase inhibitor as anti-influenza agent
		-human SIRT5 lysine deacylase inhibitor as anticancer drug
		-antitumor and anti-inflammatory compound, protease inhibitor as cancer treatment
3	(36−38)	-AT1 receptor antagonist
		-β-secretase inhibitor
		-inhibitor of the MDM2-p53
4	(39−41)	-inhibitor of eicosanoid biosynthesis as anticancer drug
5	(39,42)	-inhibitor of eicosanoid biosynthesis as anticancer drug
6	(36,39,43)	-thromboxane A2 receptor antagonist
7	(36,39,44)	-thromboxane A2 receptor antagonist
8	(39,45)	-viral neuraminidase inhibitor as anti-influenza agent
9	(46)	-HCV NS3 protease inhibitor
10	(33,36,47,48)	-neuraminidase inhibitor as anti-influenza agent
		-human SIRT5 lysine deacylase inhibitor as anticancer drug
		-antitumor and anti-inflammatory compound, protease inhibitor as cancer treatment
11	(49)	-HCV NS3 protease inhibitor
12	(39)	-β-secretase inhibitor
13	(36,50)	-MEK inhibitor-LAT2
14	(33,39)	-viral neuraminidase inhibitor as anti-influenza agent
15	(36)	-aldose reductase inhibitor
16	(51)	-BACE1 inhibitor
17	(52)	-AKR1C3 inhibitor
18	(36)	-NMDA receptor antagonist
19	(39,42)	-inhibitor of eicosanoid biosynthesis/cyclooxygenase as anticancer drug
20	(36)	-NMDA receptor antagonist
21	(53)	-stimulator of transport properties of human MRP2 (sulfanitran)
22	(4,36,39)	-antibacterial agent (sulfadiazine and sulfanilamide)
		-LTE4 receptor antagonist
		-beta3-adrenergic receptor agonist
		-antibacterial agent to improve pilicide activity against uropathogenic *E. coli*
23	(36,54)	-NMDA antagonist
24	(33,47,55)	-human SIRT5 lysine deacylase inhibitor
		-oseltamivir phosphate anti-influenza medication
25	(36,39)	-antibacterial agent (sulfadiazine and sulfanilamide)
		-LTE4 receptor antagonist
26	(36,56,57)	-modulator of the oral bioavailability of acidic drug candidates
		-MEK inhibitor
		-LAT1 binder to preserve LAT1 transport for treating brain cancer
27	(36,39)	-agonist and antagonist of GABA receptors (muscimol)
		-agonist and antagonist of the ionotropic glutamate AMPA receptor
		-agonist and antagonist of NMDA receptor
28	(39,58)	-AT1 antagonist
29	(4,36,39)	-nonpeptidic AT1 receptor antagonists (losartan), antibacterial, antiasthmatic
		-anticancer, antifungal, antihypertensive, antimalarial, antitubercular, or antiviral properties
		-based on preclinical studies, (i) inflammatory kinases inhibitor as obesity treatment and (ii) protein tyrosine phosphatase 1B inhibitor and GPR40 receptor inhibitor as antidiabetic treatment
		-antibacterial agent to improve pilicide activity against uropathogenic *E. coli*
30	(36)	-NMDA receptor antagonist
Reference Molecule		
Carboxylic Acid		
31	(4,36,39)	-antibacterial agent (sulfadiazine and sulfanilamide)
		-LTE4 receptor antagonist
		-beta3-adrenergic receptor agonist
		-antibacterial agent to improve pilicide activity against uropathogenic *E. coli*
32	(59,60)	-NMDA receptor agonist
33	(36)	-activator of the glutamate receptor (saclofen)
34	(36)	-NMDA receptor antagonist
35	(39,51)	-β-secretase inhibitor
		-BACE1 inhibitor
36	(40)	-GABA aminotransferase inhibitor
		-noncarboxylate aldose reductase inhibitor
37	(37, 43)	-viral neuraminidase inhibitor as anti-influenza agent
38	(40)	-GABA aminotransferase Inhibitor
		-noncarboxylate aldose reductase inhibitor
39	(61)	-CYP3A4Moderate inhibitor
		-MDM2-p53 Inhibitor
40	(36,39)	-antibacterial agent (sulfadiazine and sulfanilamide)
		-LTE4 receptor antagonist
		-beta3-adrenergic receptor agonist
		- xcr2 receptor antagonist
41	(36)	-AKR1C3 inhibitor
42	(36,39)	-pparg agonist (pioglitazone)
		-GPR40 agonist
43	(36,39)	-NS3 protease inhibitor (paritaprevir)
		-Pparα inhibitor
		-EP3 inhibitor
44	(36,39)	-GPR40 agonist
45	(36)	-AT1 receptor antagonist
46	(36,39)	-analogue of neurotransmitters GABA and glutamic acid (phaclofen)
47	(36)	-AT1 receptor antagonist
48	(36,39)	-antibacterial agent (sulfadiazine and sulfanilamide)-LTE4 receptor antagonist-beta3-adrenergic receptor agonist
49	(36,39)	-analogue of neurotransmitters GABA and glutamic acid (phaclofen)
50	(36,39)	-brain-penetrant EP1 receptor antagonist
51	(36)	-glutamate receptors activators (saclofen)
52	(39)	-brain-penetrant EP1 receptor antagonist
53	(39)	-brain-penetrant EP1 receptor antagonist
54	(36,62)	-AT1 receptor antagonist-PKC inhibitor

a EP1: prostaglandin E receptor 1, AT1: angiotensin II type 1 receptor, GPR40: G-protein receptor 40, LTE4: cysteinyl leukotriene receptor, GABA: g-aminobutyric acid, MEK: MAP/ERK kinase, LAT1: large neutral amino acid transporter 1, NMDA: *N*-methyl-d-aspartic acid receptors, the PPARg: peroxisome proliferator activated receptor g, NA: neuraminidase, PKC: protein kinase C, AKR1C3: aldo-keto reductase 1C3, and MRP2: multidrug resistance protein 2.

## Results and Discussion

To assess the broader applicability of the AED tool beyond the limited examples documented in the literature,^15−17,23,24^ AED values were evaluated for a comprehensive set of 54 experimentally validated nonclassical carboxylic acid bioisosteres. Figure 1 depicts the 2D structures of these moieties, and Table 1 summarizes the medicinal application(s) of each experimentally studied bioisosteric replacement, along with the corresponding reference(s).

### Effect of *R* and *S* Enantiomerism on the AED Values

*R* and *S* enantiomerism involves a change in the molecular geometry, which in turn leads to alterations in atomic electron populations and volumes. The purpose of this section is to evaluate the changes in AED values due to *R* and *S* enantiomerism in the 11 chiral bioisosteric moieties considered in this study (see Figure 1).

**Figure 2:**
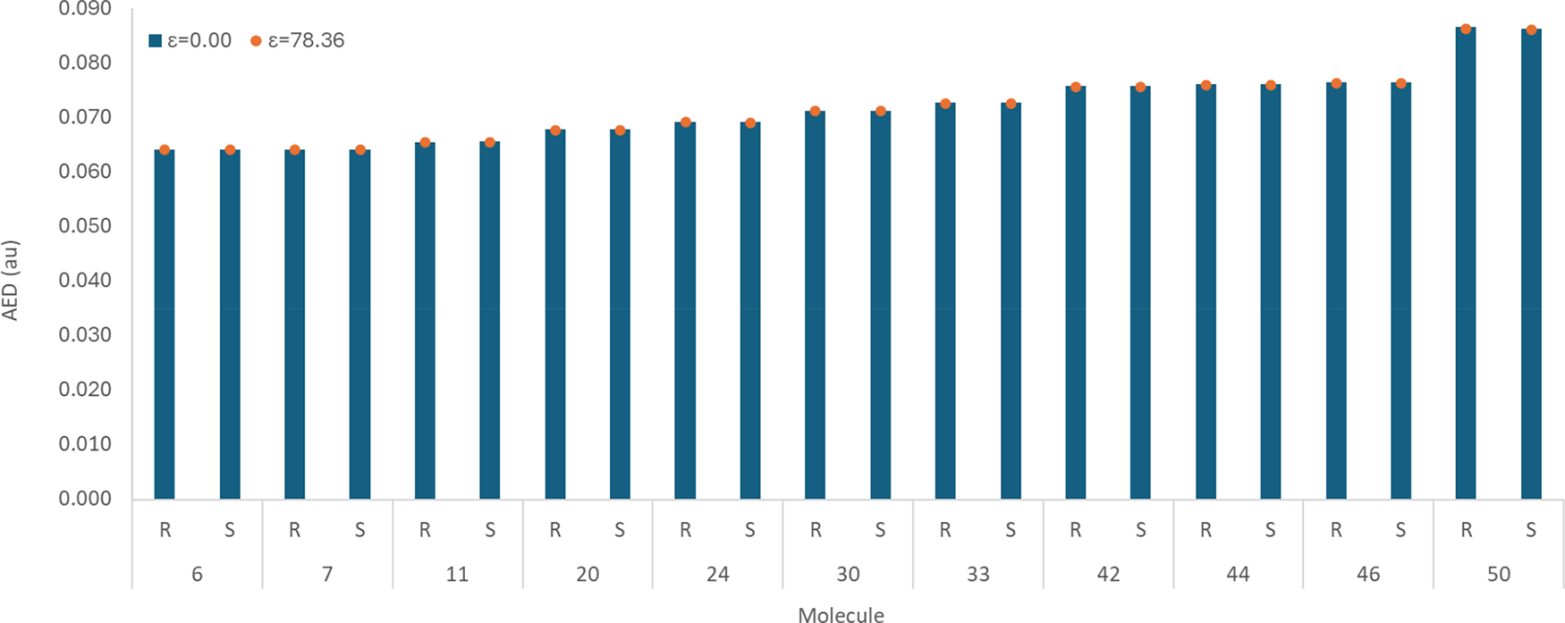
AED values of the *R* and *S* enantiomers of the 11 chiral bioisosteric moieties. The values are reported, at an isodensity value of 0.001 au, in the gas phase (ε = 0.00) and under implicit water solvation (ε = 78.36).

Figure 2 illustrates that the *R* and *S* enantiomers of the 11 chiral bioisosteric moieties of carboxylic acid have comparable AED values, irrespective of the molecule. The differences in the AED values do not exceed ∼0.23%. This is smaller than the differences observed for hydrogen atom rotation in a hydroxyl group, which shows a 0.42% variation.^17^ This 0.23% is even smaller than the differences observed in previous studies for the *cis* and *trans* constitutional isomers of an amide capped with a methyl group (0.52%) and the 1,4- and 1,5-positional isomers of a triazole capped with a methyl group (1.32%).^63^ Under both gas phase (ε = 0.00) and water (ε = 78.36), the *R* and *S* enantiomers have nearly identical AED values. In the gas phase, the AED values are identical for eight molecules, with deviations of 0.14%, 0.15%, and 0.23% for molecules 24, 11, and 50, respectively. Similarly, under water solvation, the AED of the*R* and *S* enantiomers are identical for all molecules except molecule 50 (with 0.12% difference) and molecule 24 (with 0.14% difference). Overall, the *R* and *S* enantiomers of these relatively small molecules have a maximum difference of 0.23%, regardless of the medium. Therefore, for the remainder of this work, only the *S* enantiomer will be considered.

### AED Values of 54 Carboxylic Acid Bioisosteres in the Gas Phase

The AED values of the carboxylic acid bioisosteric moieties range from 0.0586 to 0.0947 au, with a 62% difference between the minimum and maximum values. The presence of electronegative elements (*e.g.*, F, N, and O) and their abundance within a given bioisostere typically increase the AED value, unless cancellation effects occur. For instance, a moiety with a −CF_3_ group attached to a carbon bearing an alcohol group (molecule 50, 0.0864 au) has a lower AED value compared to a −CF_3_ group attached to a carbon with two alcohol groups, a geminal diol (molecule 52, 0.0892 au). Also, while difluorinated groups have high AED values, the trifluorinated moieties have the highest AED values among all the studied bioisosteres (see Figure 1 and Table 2). It is important to keep in mind that conformational changes can also influence the AED values.^63,64^

**Figure 3:**
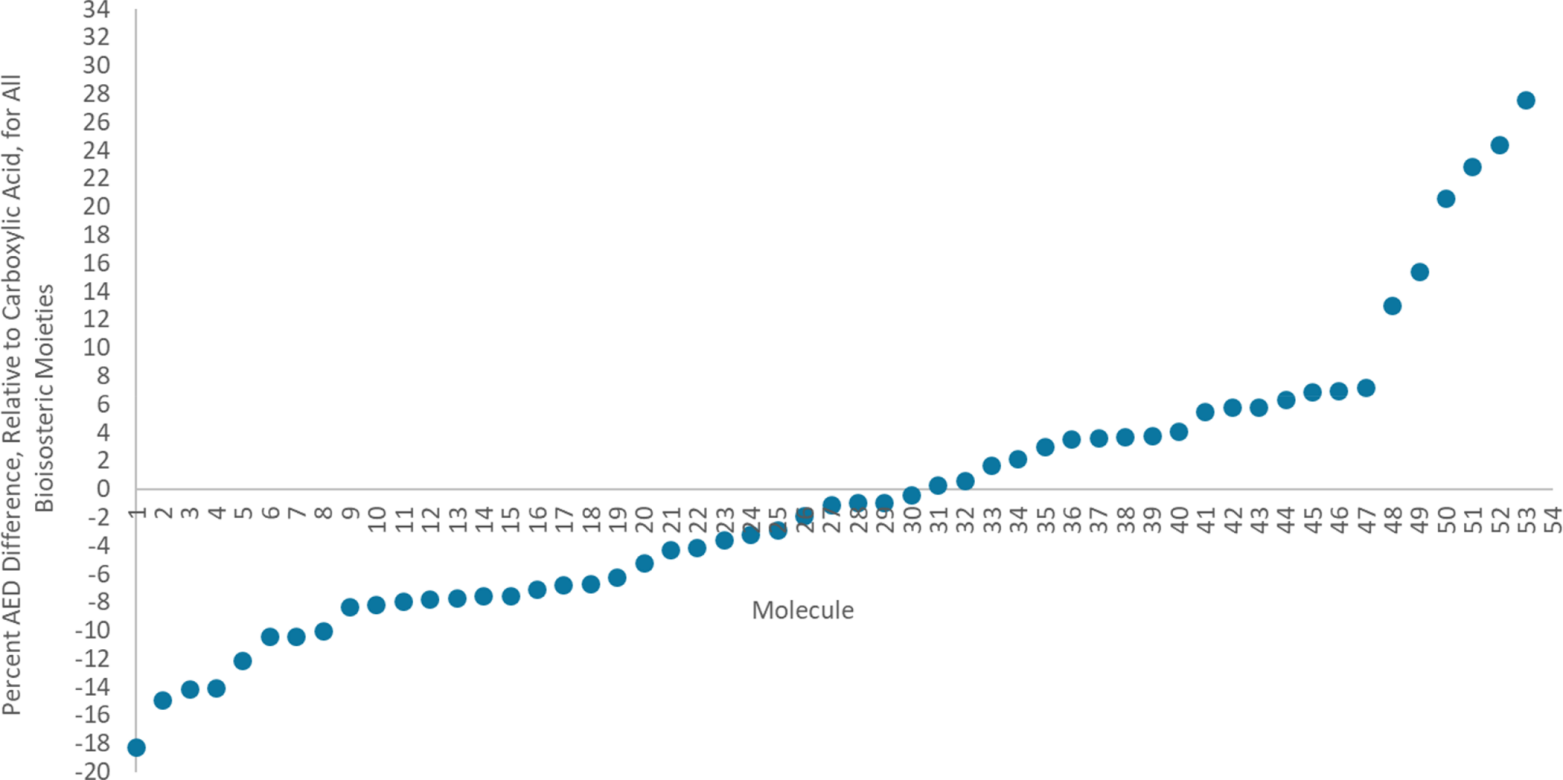
Percent AED differences (at an isodensity value of 0.001 au) between each of the 54 bioisosteric moieties considered in this study and the carboxylic acid reference moiety, in the gas phase.

Figure 3 shows the percent AED differences (at an isodensity value of 0.001 au) between the reference moiety, carboxylic acid (0.0717 au), and each of the 54 bioisosteric moieties capped with a methyl group. A negative percent AED difference indicates that the bioisosteric moiety has a lower AED value compared to carboxylic acid, while a positive value indicates a higher AED value.

The percent AED differences, relative to carboxylic acid, for all bioisosteric moieties range from −18.27 to +32.15%. Thus, an upper range of *ca.* 32% for all 65 bioisosteric moieties (including the 11 chiral molecules) serves as a reasonable threshold for identifying potential bioisosteric moieties of carboxylic acid. That is, in a drug design study, if a potential bioisostere has an AED difference greater than 32% compared to carboxylic acid (irrespective of whether it is an overestimation or an underestimation by 32%), it can be excluded from further analysis. This range implicitly covers bioisosterism, enantiomerism, and conformational changes. Furthermore, if moieties with highly electron-withdrawing groups, such as a trifluoro group, were to be ignored, a more conservative threshold of 15% covers the majority of the bioisosteres. These suggested thresholds are reliable not only because they are based on experimentally validated moieties, but also because they are supported by counterexamples. For example, the AED of the moiety in molecule 1 is 62% smaller than that of Molecule 54, and no references identify these two as bioisosteres of one another. Similar observations have been made with the furan and sulfonamide bioisosteric moieties of carboxylic acid.^16^ While the AED values of these moieties deviate by no more than ± 20% from the AED of the reference carboxylic acid moiety, their AED values differ from each other by 34%, with no evidence in the literature supporting their bioisosterism.^16^ This AED tool is distinguished for bioisosterism, as other properties such as electron populations,^15−17,23,63^ volumes,^15−17,23,63^ charges,^15−17,23,63^ geometries,^64,65^ energies,^64,65^ and in some cases, electrostatic potential maps^15−17,23^ fail to depict the similarities among bioisosteric moieties.

### Effect of Implicit Solvation on the AED Values of 54 Carboxylic Acid Bioisosteres

In biological systems, drugs and their target proteins typically exist in an intracellular aqueous environment, *e.g.*, cytoplasm. Solvation can significantly affect the dynamics of the systems, molecular interactions, and binding affinities,^66^ including interactions that involve bioisostere-containing drug molecules. This section explores the effect of implicit water solvation on the AED values to evaluate whether the AED tool remains effective for assessing bioisosterism in the presence of the most ubiquitous solvent in biological systems.

**Figure 4:**
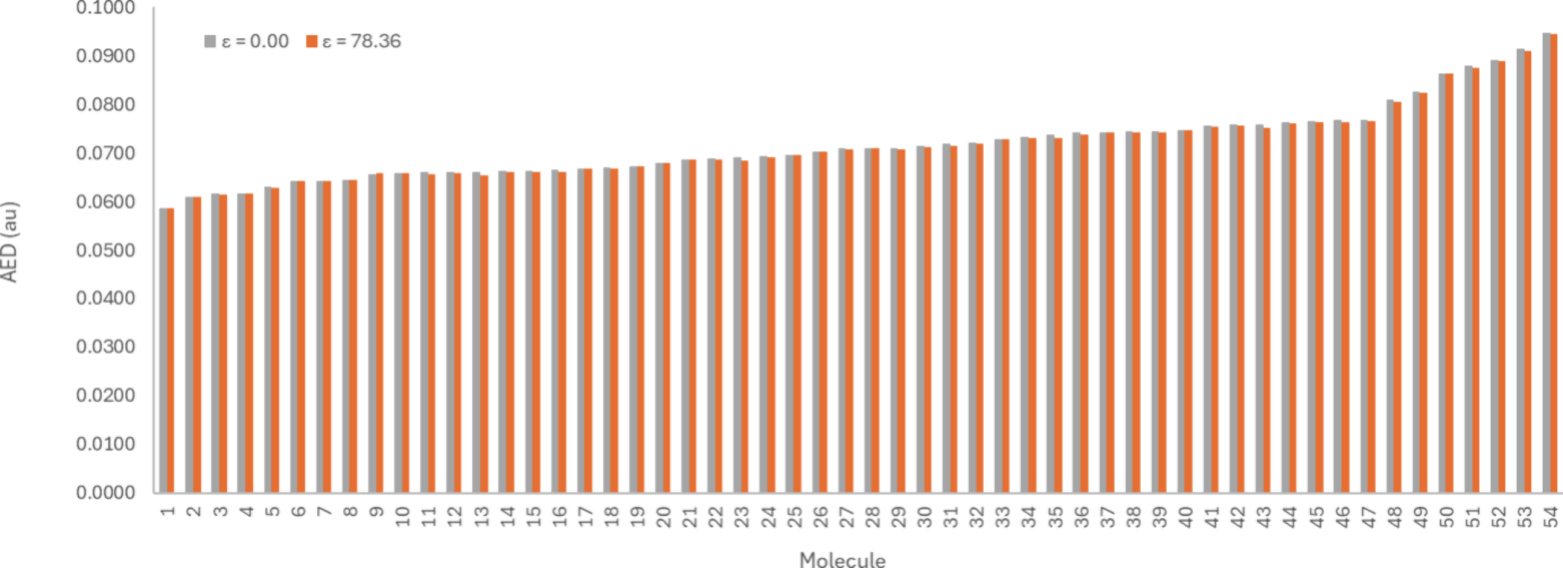
AED values, at an isodensity of 0.001 au, for the 54 nonclassical bioisosteric moieties considered in this study, calculated using two different dielectric constants: ε = 0.00 (gas phase), and ε = 78.36 (water).

As shown in Table 2 and depicted in Figure 4, the AED values of the bioisosteric moieties are generally either equal or slightly smaller under solvation (ε = 78.36) compared to the gas phase (ε = 0.00), except in molecule 9, where the AED is slightly higher upon solvation. Consequently, the trends across the moieties remain consistent. The span of the AED differences of the bioisosteres (relative to carboxylic acid) remains −18 to +32%, as observed in the gas phase. This confirms that the AED tool can be equally applicable for evaluating bioisosterism in molecules, regardless of whether they are in vacuum or in water.

**Table 2:** Volume, Electron Population, and AED (All at an Isodensity of 0.001 au) for the 54 Bioisosteric Moieties Considered in This Study, Evaluated in the Gas Phase (ε = 0.00) and Water (ε = 78.36)

	**ε = 0.00**			**ε = 78.36**		
**molecule no.**	**volume (au)**	**electron population (au)**	**AED (au)**	**volume (au)**	**electron population (au)**	**AED (au)**
1	732.09	42.88	0.0586	732.48	42.90	0.0586
2	1029.06	62.75	0.0610	1029.56	62.77	0.0610
3	567.09	34.91	0.0616	568.73	34.94	0.0614
4	759.97	46.80	0.0616	760.49	46.80	0.0615
5	869.46	54.76	0.0630	870.80	54.76	0.0629
6	792.08	50.84	0.0642	792.41	50.84	0.0642
7	790.86	50.83	0.0642	791.40	50.84	0.0642
8	1623.23	104.71	0.0645	1626.58	104.77	0.0644
9	529.05	34.96	0.0657	531.63	35.02	0.0659
10	339.41	22.29	0.0658	339.04	22.28	0.0657
11	1441.75	94.71	0.0660	1443.45	94.76	0.0656
12	528.54	34.97	0.0661	531.54	35.00	0.0658
13	589.29	38.89	0.0662	595.46	38.88	0.0653
14	1339.31	88.72	0.0662	1343.61	88.79	0.0661
15	647.81	42.93	0.0662	649.92	42.96	0.0661
16	883.94	58.87	0.0666	893.10	58.92	0.0660
17	760.73	50.89	0.0668	761.71	50.90	0.0668
18	1239.08	82.79	0.0669	1238.38	82.78	0.0668
19	934.09	62.77	0.0672	934.53	62.77	0.0672
20	748.53	50.86	0.0679	750.16	50.88	0.0678
21	1169.35	80.79	0.0686	1179.39	80.86	0.0686
22	1497.28	102.69	0.0687	1495.27	102.68	0.0687
23	832.19	57.19	0.0691	835.67	57.23	0.0685
24	812.48	56.28	0.0694	814.46	56.29	0.0691
25	816.39	56.83	0.0696	817.00	56.84	0.0696
26	440.68	30.98	0.0703	441.63	31.01	0.0702
27	606.53	42.97	0.0708	608.71	43.00	0.0706
28	716.75	50.87	0.0710	718.11	50.88	0.0709
29	493.13	35.00	0.0710	494.71	35.02	0.0708
30	713.26	50.91	0.0714	714.79	50.93	0.0713
Reference Molecule	320.97	23.00	0.0717	323.49	23.06	0.0713
Carboxylic Acid						
31	985.30	70.82	0.0719	990.36	70.81	0.0715
32	706.00	50.90	0.0721	708.76	50.94	0.0719
33	561.25	40.90	0.0729	562.34	40.92	0.0728
34	668.37	48.94	0.0732	671.42	48.99	0.0730
35	690.79	50.98	0.0738	697.60	51.02	0.0731
36	551.40	40.91	0.0742	554.98	40.98	0.0738
37	873.11	64.86	0.0743	874.31	64.88	0.0742
38	872.29	64.84	0.0743	873.19	64.86	0.0743
39	497.36	36.99	0.0744	499.31	37.02	0.0741
40	954.78	71.20	0.0746	954.28	71.22	0.0746
41	568.92	43.00	0.0756	571.74	43.03	0.0753
42	776.85	58.89	0.0758	778.12	58.90	0.0757
43	829.39	62.90	0.0758	836.54	62.94	0.0752
44	668.36	50.94	0.0762	670.11	50.96	0.0760
45	561.42	43.01	0.0766	564.62	43.06	0.0763
46	422.26	32.37	0.0767	424.19	32.39	0.0764
47	663.31	50.94	0.0768	666.09	50.98	0.0765
48	505.86	40.97	0.0810	509.37	41.02	0.0805
49	488.27	40.37	0.0827	489.87	40.39	0.0824
50	566.72	48.97	0.0864	567.55	48.97	0.0863
51	466.03	41.02	0.0880	469.02	41.07	0.0876
52	638.77	56.96	0.0892	640.54	56.99	0.0890
53	514.17	47.02	0.0914	516.79	47.06	0.0911
54	773.82	73.29	0.0947	775.98	73.32	0.0945

**Figure 5:**
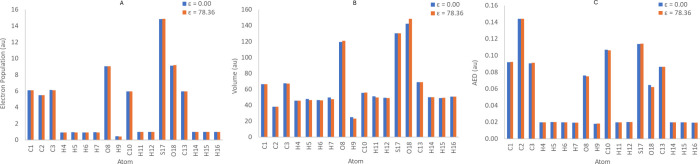
Electron population (A), volume (B), and AED (C) per atom of molecule 19.

Solvation induced conformational changes, whether subtle around functional groups like hydroxyl (OH) or more extensive across the molecule, both known to influence AED values.^63,64^ Therefore, the reported AED values implicitly account for these conformational changes. It is worth noting that a minor change in the AED of the bioisosteric moiety (see, *e.g.*, molecule 19 in Figure 4) is not necessarily associated with equally minor deviations in the AEDs (or even volumes and electron populations) of its atomic constituents. For example, Figure 5 shows that, upon solvation, there are relatively greater changes in the atomic properties of the bioisosteric moiety in molecule 19, especially the volumes of atoms H7, H9, and O18. However, the compensation at the atomic level results in a net AED difference of 0.05% in the bioisosteric moiety.

Overall, the deviations in the AED values for a given moiety seem to be less influenced by changes in the dielectric constant of the medium compared with conformational changes. The maximum difference of *ca.* 1.30% between the gas phase and water is about one-third of the 3.5% variation observed in the AEDs of different conformers of a molecule,^64^ yet it is approximately double the AED variations of 0.42% resulting from simpler rotations, such as the rotation of a single hydrogen atom in the hydroxyl group of a carboxylic acid.^17^

## Conclusions

The AED tool has been proven to be effective in evaluating the bioisosterism of an extended set of 54 (65 if the R and S enantiomers of the chiral moieties are counted) experimentally reported nonclassical carboxylic acid bioisosteres. The *R* and *S* enantiomers in such small molecules show negligible AED differences, under 0.23%. The percent AED difference between each of these moieties with respect to carboxylic acid does not exceed 32%. It is, therefore, suggested that 32% can serve as a reasonable threshold to confirm the bioisosterism of potential moieties with carboxylic acid, irrespective of whether the AED is overestimated or underestimated. Using the AIMALL software, which is based on the QTAIM theory, the AED trends in the water-solvated bioisosteric moieties are similar to those in the gas phase. This study advances the understanding of AED in bioisosteric moieties by demonstrating its consistency across solvation and enantiomerism, reinforcing its reliability as a predictive tool in molecular design.

## Supporting Information Available

*XYZ* coordinates of the 54 molecules (both *R* and *S* enantiomers, as applicable) are included, along with a list of the molecule numbers used in the manuscript and their corresponding molecule names associated with the *XYZ* coordinates (PDF)
