# Supplementary material for: Quantum Evaluation of a Comprehensive Set of Carboxylic Acid Bioisosteres: Gas versus Solvated Phases
Source: ACS Omega. 2025 Apr 25;10(17):17684–93. doi: 10.1021/acsomega.4c11714 (PMC12059892; doi:10.1021/acsomega.4c11714)
Supplement: Supplementary file 1 — ao4c11714_si_001.pdf [file ao4c11714_si_001.pdf]

Quantum Evaluation of a Comprehensive Set of Carboxylic Acid Bioisosteres: Gas versus Solvated Phases

Alaa MA Osman[1], Alya A. Arabi[1*]

[1]College of Medicine and Health Sciences, Department of Biochemistry and Molecular Biology, United Arab Emirates University, AlAin, P. O. Box: 15551, United Arab Emirates.

[*]Corresponding author: alya.arabi@uaeu.ac.ae; alya.arabi@dal.ca

The table below lists the molecule numbers used in the manuscript, along with the corresponding molecule names associated with their XYZ coordinates (provided below). The XYZ coordinates for both the R and S enantiomers of molecules 6, 7, 11, 20, 24, 30, 33, 42, 44, 46, and 50 are provided.

Molecule No.	Molecule Name
1	5-methylfuran
2	Boronic acid 1
3	Imidazole
4	Oxetan-3-ol
5	Thietan-3-ol
6	Cyclopentane-1,2-dione_R<br>Cyclopentane-1,2-dione_S
7	Cyclopentane-1,3-dione_R<br>Cyclopentane-1,3-dione_S
8	Sulfonate ester
9	Acyl cyanamide
10	Boronic acid 2
11	Acyl sulfinamide_R<br>Acyl sulfinamide_S
12	1,2,4-triazole
13	Hydroxamic acid 2
14	Sulfones 2
15	1-hydroxypyrazole
16	BACE1 carboxylic bioisosteres 2
17	Hydroxytriazole
18	4-hydroxyquinoline-2-one
19	Thietan-1-oxide-3-ol
20	Tetramic acid_R<br>Tetramic acid_S
21	Aryl sulfonamides
22	Acyl sulfonamides 2
23	Squaric acid 2
24	Phosphonate monoester_R

	Phosphonate monoester_S
25	Sulfonamides (with methyl)
26	Hydroxymic acid 1
27	3-hydroxyisoxazole
28	Tetrazolone
29	Tetrazole
30	Tetronic acid_R<br>Tetronic acid_S
31	Acyl sulfonamides 1
32	Isothiazole
33	Sulfinic acid_R<br>Sulfinic acid_S
34	Squaric acid
35	2-thioxo-1,3,4-oxadiazole
36	Sulfones 1
37	2,6-difluorophenols 2
38	2,6-difluorophenols 1
39	Alpha-keto acid
40	Sulfonylureas
41	Hydroxyfurazan
42	2,4-thiazolidinedione_R<br>2,4-thiazolidinedione_S
43	Acyl sulfonamides p1
44	2,4-oxazolidinedione_R<br>2,4-oxazolidinedione_S
45	5-oxo-1,2,4-oxadiazole
46	Phosphinic acid_R<br>Phosphinic acid_S
47	5-oxo-1,2,4-thiadiazole
48	Sulfonamides
49	Phosphonic acids
50	2,2,2-triflouroethan-1-ol_R<br>2,2,2-triflouroethan-1-ol_S
51	Sulphonic acid
52	2,2,2-triflouromethayl-ketones (hydrated)
53	Trifluoromethyl ketone
54	Trifluoromethyl sulfonamide

Below are the XYZ coordinates of the 54 molecules.

C	-0.72458806	-0.73705139	-0.30293155
C	-0.33000004	-2.18730829	0.03270995
H	-0.49235524	-2.36909330	1.07458086
H	-0.92713587	-2.86207254	-0.54437063
H	0.70365281	-2.33840505	-0.19887493
O	2.13604754	-0.93994956	-0.13149462
N	2.05746812	1.43345857	0.30506533
N	0.90306873	2.21784217	0.08930309
N	-0.15978147	1.54464515	0.19099594
N	0.09577613	0.18996118	0.48988009
H	-0.56223286	-0.55526638	-1.34480246
H	-1.75824091	-0.58595463	-0.07134667

Tetrazolone

C	-0.36381421	0.52211996	-0.27694360
C	1.61765984	1.93698157	0.10925562
C	0.27426919	2.66173836	0.06073317
H	-0.77685533	-0.35790764	0.17010053
H	2.01735914	1.99449243	1.10013047
H	2.35499275	2.31157759	-0.56965381
C	1.17308697	0.53235133	-0.23084000
O	1.92631675	-0.44953406	-0.45913045
O	0.11485674	3.87748100	-0.22233586
C	-0.81392251	0.59239228	-1.74801951
H	-0.46638771	-0.27508668	-2.26917190
H	-1.88228912	0.63112671	-1.79265625
H	-0.40582829	1.46996239	-2.20434156
O	-0.79044422	1.73330474	0.40087317

Tetronic acid_R

C	1.47830000	1.64280000	-0.52050000
C	0.71840000	0.74060000	0.45400000
H	1.06190000	0.92440000	1.47200000
C	0.94290000	-0.71070000	0.09090000
O	1.99090000	-1.30930000	0.05220000
C	-0.44180000	-1.24490000	-0.21340000
C	-1.38310000	-0.08420000	-0.01160000
O	-2.58080000	-0.13120000	-0.16370000
O	-0.70280000	1.00340000	0.36020000
H	1.12180000	1.46350000	-1.53490000
H	1.31030000	2.68670000	-0.25560000
H	2.54400000	1.42090000	-0.46540000
H	-0.49170000	-1.59480000	-1.24460000
H	-0.69130000	-2.05510000	0.47180000

Tetronic acid_S

C	0.75387844	0.14025192	-1.39912802
C	2.16156142	-0.48321894	-1.59908704
C	2.66021421	0.83811943	-2.24382046
H	-0.09992758	-0.49675531	-1.29844507
H	0.73041853	0.80025718	-0.55725945
H	3.50763578	0.82389413	-2.89694704
H	2.86959002	1.58336072	-1.50512080
O	2.11131930	-1.52722444	-2.57501134
H	3.00461005	-1.78594594	-2.81311808
C	2.91233935	-1.06940686	-0.38899769
H	3.89571074	-1.36598069	-0.68887213
H	2.98214616	-0.32897221	0.38027699
C	2.14732810	-2.29442250	0.14550626
H	1.16395671	-1.99784867	0.44538070
H	2.07752129	-3.03485715	-0.62376842
H	2.66897250	-2.70170891	0.98628262
S	1.03020465	1.02682132	-2.90481419
O	0.73907449	0.30985966	-4.15418738

Thietan-1-oxide-3-ol

C	0.83700000	0.13090000	-1.45330000
C	2.21170000	-0.49330000	-1.59490000
C	2.71620000	0.79290000	-2.22010000
H	-0.00340000	-0.52760000	-1.38230000

C	2.02075819	0.08238576	-3.96696623
C	2.69580906	-0.97009349	-4.58684125
C	0.84027254	0.55593543	-4.54129683
C	2.17442385	-1.52267424	-5.76460639
H	3.60297682	-1.35161503	-4.16685557
C	0.36423536	-0.03394038	-5.72017506
H	0.30361536	1.36184334	-4.08587227
N	1.03554606	-1.04608494	-6.29476351
H	2.68697415	-2.32979712	-6.24496545
H	-0.54021046	0.32430166	-6.16575377

Sulfones 2

S	-0.39593344	1.58054495	0.16485161
O	0.88483003	2.29517668	0.25796774
O	-1.50200722	2.53383651	0.33086416
C	-0.48040599	0.34516476	1.44356197
H	-1.41291613	-0.17515093	1.37576511
H	0.32491447	-0.34891659	1.32269001
H	-0.40399474	0.81694563	2.40089374
O	-0.51519211	0.84421407	-1.32930173
H	0.20733840	0.22148687	-1.43774759

Sulphonic acid

C	-0.37040000	0.49820000	-0.27110000
C	1.61400000	1.94150000	0.09320000
C	0.27140000	2.68850000	0.06290000
H	-0.79810000	-0.38500000	0.15560000
H	2.02810000	2.00770000	1.07760000
H	2.34170000	2.30420000	-0.60240000
C	1.16480000	0.51740000	-0.22290000
O	1.92040000	-0.46570000	-0.43770000
O	0.10670000	3.90480000	-0.21440000
C	-0.80190000	0.59390000	-1.74630000
H	-0.45320000	-0.26750000	-2.27650000
H	-1.86940000	0.63990000	-1.80340000
H	-0.38300000	1.47590000	-2.18390000
N	-0.79210000	1.72720000	0.43730000
H	-0.79370000	1.62060000	1.43160000

Tetramic acid_R

C	1.48080000	1.64490000	-0.52430000
C	0.72140000	0.74650000	0.45400000
H	1.06580000	0.93320000	1.47120000
C	0.94300000	-0.70620000	0.09530000
O	1.99100000	-1.30520000	0.05840000
C	-0.44030000	-1.24320000	-0.20690000
C	-1.38880000	-0.08800000	-0.01100000
O	-2.59050000	-0.14370000	-0.16440000
N	-0.71330000	1.01100000	0.35940000
H	1.12320000	1.46230000	-1.53770000
H	1.31370000	2.68970000	-0.26270000
H	2.54650000	1.42250000	-0.46950000
H	-0.48850000	-1.59760000	-1.23650000
H	-0.68720000	-2.05200000	0.48080000
H	-1.12510000	1.87050000	0.53950000

Tetramic acid_S

C	0.62146579	1.99795138	0.02008820
N	-0.41180032	1.27438726	-0.19497498
N	-0.00230868	-0.06708085	-0.08333320
H	-0.30510910	-0.45417561	0.78756838
N	1.41318142	-0.00494569	-0.13130065
N	1.80198772	1.15031118	0.07675115
C	0.61683020	3.52907718	0.18511027
H	-0.18661592	3.94680969	-0.38486682
H	0.48740671	3.77608169	1.21813415
H	1.54647898	3.92817433	-0.16327833

Tetrazole

C	1.50668945	0.10127195	0.18996568
H	2.78209717	1.61725010	-0.35911342

C	2.31020000	-0.13000000	-0.07780000
C	1.07890000	0.67530000	0.34230000
S	-0.40820000	-0.07500000	-0.37610000
O	-0.73880000	-1.25450000	0.34420000
O	-1.46980000	0.87160000	0.16560000
H	3.20380000	0.32080000	0.35390000
H	2.21240000	-1.15560000	0.27830000
H	2.39150000	-0.12900000	-1.16470000
H	0.99760000	0.67430000	1.42920000
H	1.17670000	1.70080000	-0.01380000
H	-2.32160000	0.55510000	-0.16530000

Sulfinic acid_S

S	2.57468165	0.78653596	0.02189034
O	1.97571093	2.12714249	0.08298361
O	4.03919477	0.90519110	-0.00720327
O	2.05192340	0.00565551	-1.35863475
C	2.44651148	0.75693764	-2.50962364
H	2.01040732	1.73301887	-2.46514228
H	3.51280778	0.84332917	-2.53080638
C	1.96444699	0.03684429	-3.78268270
H	0.89815069	-0.04954725	-3.76149996
H	2.40055115	-0.93923695	-3.82716406
C	2.38938800	0.84591734	-5.02220919
H	1.95328384	1.82199858	-4.97772784
H	3.45568430	0.93230888	-5.04339194
C	1.90732351	0.12582399	-6.29526826
C	2.73152707	-0.81978246	-6.92011935
C	0.64444126	0.41614550	-6.82890092
C	2.29284745	-1.47506908	-8.07860181
H	3.69576599	-1.04144883	-6.51267940
C	0.20576177	-0.23914090	-7.98738355
H	0.01514468	1.13813902	-6.35181481
C	1.02996546	-1.18474710	-8.61223484
H	2.92214461	-2.19706157	-8.55568873
H	-0.75847751	-0.01747515	-8.39482302
H	0.69502387	-1.68507292	-9.49676194
C	2.08351606	-0.14862640	1.45458979
H	2.41845698	0.35169820	2.33911784
H	1.01721976	-0.23501794	1.47577254
H	2.51962022	-1.12470763	1.41010844

Sulfonate ester

S	2.65092427	0.73339484	0.01414715
O	2.25726779	2.14900408	0.04259393
O	4.11702489	0.63278962	0.00183037
C	2.01184686	-0.08928801	1.45746728
H	0.94439471	-0.01603843	1.46643500
H	2.29846392	-1.11997791	1.43675549
H	2.40851743	0.37361825	2.33682515
C	1.99104238	-0.03667352	-1.44870986
H	0.92359024	0.03657606	-1.43974214
H	2.37520689	0.45786055	-2.31632364
H	2.27765944	-1.06736343	-1.46942165

Sulfones 1

S	2.62089222	0.69246200	0.04179662
O	2.23326982	2.10875027	0.10180125
O	4.08646040	0.58465051	0.05631444
C	1.94878736	-0.16674600	1.44836575
H	0.88172289	-0.08824962	1.43779546
H	2.23101108	-1.19793030	1.40467703
H	2.32961003	0.26945172	2.34814664
C	1.98737414	-0.03317534	-1.45503512
H	2.26959786	-1.06435964	-1.49872384
H	0.92030968	0.04532104	-1.46560539
C	2.56885810	0.71018438	-2.67195449
H	3.63592259	0.63168802	-2.66138421
H	2.28663435	1.74136869	-2.62826578

H	-1.91070000	-2.09990000	-0.10730000
H	-0.87710000	1.18270000	1.69380000

Phosphonate monoester_S

P	0.47255765	0.12068597	-0.15751086
C	1.10632539	0.53623583	1.49719660
H	1.36904482	1.57300897	1.52850081
H	0.35112837	0.33742037	2.22866763
H	1.97140270	-0.05741507	1.70724387
O	1.67946130	0.43841909	-1.32649728
H	1.34516622	0.21922795	-2.19931001
O	0.10308083	-1.33738527	-0.20153569
O	-0.90994908	1.06941779	-0.49319388
H	-1.58750902	0.89104130	0.16307920

Phosphonic acids

C	-1.10289125	0.94074044	0.52937862
C	-0.11662581	0.44215411	1.60355401
C	0.27254429	1.88691582	1.82529934
C	-0.74716861	2.26133130	0.73206594
O	1.07352367	2.50769712	2.57137788
O	0.21692038	-0.67237150	2.08329367
N	-2.05583181	0.26988828	-0.36659106
H	-1.59894350	0.03707709	-1.22510841
H	-2.39198953	-0.56503252	0.06919012
N	-1.17664111	3.53381116	0.13436335
H	-0.41783640	4.18479473	0.15523947
C	-2.30907007	4.07659040	0.89849379
H	-2.00882481	4.24200021	1.91209746
H	-2.62167932	5.00281723	0.46343135
H	-3.12099111	3.38003799	0.87615638

Squaric acid 2

C	-1.09900000	0.87040000	0.41170000
C	-0.18890000	0.43300000	1.57600000
C	0.02730000	1.90010000	1.87380000
C	-0.90140000	2.21150000	0.68390000
C	-1.41290000	3.51550000	0.04390000
H	-0.73510000	3.82590000	-0.72360000
H	-2.38060000	3.34860000	-0.38100000
H	-1.47830000	4.27800000	0.79170000
O	0.67710000	2.56950000	2.71830000
O	0.20120000	-0.65990000	2.06280000
O	-1.85690000	0.16150000	-0.57220000
H	-1.91220000	0.68740000	-1.37350000

Squaric acid

C	-1.96853311	-0.45030233	-0.06966186
C	-3.44555039	-0.71475810	0.27686823
H	-4.06895620	-0.33518229	-0.50555582
H	-3.60457568	-1.76779010	0.38047592
H	-3.68935856	-0.22504714	1.19645550
O	-1.60182407	-0.41094542	-1.27280195
N	-0.98702416	-0.24384207	1.00500754
H	-0.08942371	-0.56478089	0.70282926
O	-1.36971826	-0.94060944	2.10848407
H	-0.72873282	-0.80577824	2.81030899

Hydroxymic acid 1

C	-3.27160000	0.43190000	0.08160000
H	-2.94910000	-0.58390000	-0.01390000
H	-2.62570000	0.94880000	0.76020000
H	-4.27370000	0.45420000	0.45610000
C	-3.22310000	1.11770000	-1.29640000
H	-2.22100000	1.09530000	-1.67080000
H	-3.86900000	0.60070000	-1.97500000
S	-3.75960000	2.80740000	-1.13740000
O	-5.13600000	2.83810000	-0.62320000
O	-3.70700000	3.55110000	-2.63180000
H	-3.99640000	4.46240000	-2.54600000

Sulfinic acid_R

H	0.02631779	-0.44277871	-1.47603829
H	0.83246437	0.83819583	-0.67265506
H	3.45726538	0.76372174	-2.88230441
H	2.82755869	1.53977525	-1.49039812
O	2.19651054	-1.55747306	-2.54367135
H	3.09780820	-1.82052344	-2.74383887
C	2.92384655	-1.08098793	-0.36034972
H	3.91255362	-1.38634283	-0.63259356
H	2.97872007	-0.33980990	0.40941909
C	2.13410109	-2.29858725	0.15477549
H	1.14539402	-1.99323235	0.42701934
H	2.07922757	-3.03976529	-0.61499332
H	2.62896243	-2.70875846	1.01021213
O	1.23506563	0.92780471	-2.75712907

Oxetan-3-ol

P	0.42658399	0.08851121	-0.13964925
C	1.03547943	0.32909094	1.55851674
H	1.16958894	1.37421248	1.74462317
H	0.32420538	-0.06830416	2.25210622
H	1.97062096	-0.17719577	1.67719315
O	1.56329299	0.72360058	-1.24809599
H	1.24211737	0.59670138	-2.14383189
O	0.23797839	-1.38130084	-0.40138098
H	-0.75326747	0.72728417	-0.28938117

Phosphinic acid_R

C	1.65020000	-0.45270000	-0.18840000
P	-0.02880000	0.05730000	0.30570000
H	-0.10500000	0.12600000	1.72200000
O	-0.32970000	1.38730000	-0.26960000
O	-1.09770000	-1.01690000	-0.23790000
H	2.37400000	0.27460000	0.17960000
H	1.87190000	-1.43220000	0.23530000
H	1.70880000	-0.50540000	-1.27550000
H	-1.97030000	-0.71030000	0.04440000

Phosphinic acid_S

O	-0.37254410	2.40587629	-0.05877539
O	-1.66542617	-0.17183360	-0.88855380
O	-0.76216291	1.71567120	-1.92427653
C	-2.15864026	2.00060136	-2.04074893
H	-2.71447010	1.08778937	-1.98852380
C	-1.15428822	0.84531862	1.25320808
H	-2.20601288	1.03883790	1.21680036
H	-0.98537978	-0.13430153	1.64907964
H	-0.67974205	1.57037108	1.88090615
C	-2.43090088	2.69091150	-3.39017328
H	-3.47581750	2.90411098	-3.47732396
H	-1.87507104	3.60372348	-3.44239842
H	-2.13098220	2.04453109	-4.18838451
H	-2.45855894	2.64698176	-1.24253771
H	-1.48048537	-1.02431037	-0.48770438
P	-0.45984755	0.94915799	-0.42588976

Phosphonate monoester_R

C	3.09090000	-0.26950000	0.15480000
C	1.69780000	0.27430000	-0.16860000
O	0.70710000	-0.62660000	0.33030000
P	-0.72620000	0.00460000	-0.04300000
O	-0.78970000	0.25670000	-1.50000000
C	-2.04540000	-1.16120000	0.43010000
O	-0.92520000	1.39110000	0.75070000
H	3.84650000	0.41770000	-0.22580000
H	3.20060000	-0.36830000	1.23480000
H	3.21790000	-1.24520000	-0.31410000
H	1.57070000	1.25000000	0.30030000
H	1.58810000	0.37310000	-1.24860000
H	-1.99860000	-1.34690000	1.50310000
H	-3.01570000	-0.73390000	0.17730000

C	1.69586255	1.13996596	0.06174071
C	2.14739789	2.56206590	-0.02688700
O	-0.03162743	2.45661189	-0.21657121
N	1.08950897	3.28020805	-0.09787332
N	0.41569665	1.15806905	0.03449382
O	2.53128016	-0.01609303	0.16423478
H	2.73878469	-0.33995075	-0.71534356
C	3.60249946	3.06632596	-0.02579786
H	4.21265385	2.39368453	0.54003350
H	3.96379467	3.11519179	-1.03176899
H	3.64206201	4.04046408	0.41509865

Hydroxyfurazan

C	0.59313997	0.44008808	0.12267199
C	1.05393978	1.87072056	0.01319335
O	1.43223894	-0.71561356	0.19455126
H	1.60448917	-1.04162878	-0.69181431
N	-0.69025478	0.44945276	0.14854868
N	-1.13705366	1.76996058	-0.15330692
N	0.00624765	2.61185886	-0.01708151
C	2.51460657	2.35548153	-0.04211804
H	3.13704843	1.67492578	0.50038542
H	2.83754709	2.39918696	-1.06128400
H	2.58410305	3.32914630	0.39611383
C	-1.63970081	1.82613674	-1.53355692
H	-2.47700163	1.16774670	-1.63529136
H	-1.94272387	2.82650239	-1.76234853
H	-0.86524935	1.52505126	-2.20770265

Hydroxytriazole

C	-0.26138914	-0.59539056	0.01225174
C	0.31176662	1.60153640	0.11094456
N	-0.80379052	0.71149029	0.43488405
H	-1.65509814	0.95885920	-0.02780802
H	-0.86012249	-1.46166682	-0.17744230
N	1.03705873	-0.56123415	-0.11034700
C	0.23191186	3.13503781	-0.00566017
H	-0.00610138	3.40418482	-1.01353396
H	-0.52833547	3.50355371	0.65093715
H	1.17468894	3.56285963	0.26459874
C	1.41652925	0.85841192	-0.07460220
H	2.40961176	1.23928439	-0.19132102

Imidazole

C	-0.00564224	-0.14822303	-0.06622380
C	1.33791287	-0.11476412	-0.03497434
C	1.85694628	1.34421802	0.02202987
H	1.96573038	-0.98120952	-0.03916234
C	-0.87081621	-1.41885815	0.02629851
H	-1.09410705	-1.62438351	1.05235920
H	-1.78220946	-1.26992171	-0.51414601
H	-0.33725950	-2.24511312	-0.39503266
S	-0.61737789	1.49821953	-0.22096665
N	0.90289876	2.24398406	0.04031466
O	3.24687155	1.67815059	0.06071822
H	3.54051097	1.72049565	0.97372577

Isothiazole

C	0.90017723	0.17382850	-1.50867600
C	2.21161622	-0.49064806	-1.59153889
C	2.62158888	0.77916679	-2.21424286

C	0.81480000	2.28120000	0.10370000
H	0.44160000	2.44990000	-0.88470000
H	0.00570000	2.40610000	0.79260000
H	0.86280000	0.09350000	-0.11660000
H	1.73480000	0.71780000	1.24890000
H	2.56240000	1.14070000	-1.64040000

Cyclopentane-1,2-dione_R

C	2.25400000	-0.26600000	0.68960000
C	1.21580000	0.13680000	-0.35970000
H	1.64550000	0.05780000	-1.35820000
C	0.74110000	1.57780000	-0.10380000
C	-0.78820000	1.53010000	-0.29270000
C	-1.17510000	0.11090000	0.07560000
O	-2.22930000	-0.25100000	0.54100000
C	-0.00250000	-0.75210000	-0.24930000
O	-0.02300000	-1.95070000	-0.39770000
H	1.81210000	-0.19590000	1.68350000
H	2.57650000	-1.29100000	0.50680000
H	3.11290000	0.40200000	0.62590000
H	0.98840000	1.88370000	0.91270000
H	1.19130000	2.25910000	-0.82580000
H	-1.27580000	2.24190000	0.37340000
H	-1.04970000	1.73830000	-1.33010000

Cyclopentane-1,2-dione_S

C	-1.37730000	1.64530000	0.69670000
C	-0.83630000	0.77840000	-0.44200000
H	-1.38480000	0.98020000	-1.36210000
C	0.66380000	1.04590000	-0.64770000
C	1.40630000	-0.09270000	0.01710000
O	2.60440000	-0.23590000	0.06350000
C	0.36840000	-1.01830000	0.61340000
C	-0.94280000	-0.69110000	-0.07900000
O	-1.86750000	-1.43780000	-0.29300000
H	-1.25250000	2.69800000	0.44270000
H	-2.43540000	1.43050000	0.84580000
H	-0.82920000	1.42520000	1.61280000
H	0.89550000	1.07060000	-1.71250000
H	0.94060000	1.99260000	-0.18360000
H	0.64130000	-2.05690000	0.42630000
H	0.28030000	-0.84100000	1.68530000

Cyclopentane-1,3-dione_R

C	-0.38607253	0.47282604	-0.28946423
C	1.61085106	1.94558838	0.08183627
C	0.28249582	2.73704658	0.05163052
H	-0.82906671	-0.41193186	0.11779442
H	2.02569275	2.01277726	1.06585458
H	2.34284007	2.28817748	-0.61939464
C	1.14794143	0.50432648	-0.21954339
O	1.90555837	-0.48160261	-0.41327245
O	0.13886952	3.95697321	-0.22172100
C	-0.78362869	0.58309956	-1.77317204
H	-0.42530244	-0.27459896	-2.30313010
H	-1.84940530	0.63280041	-1.85410436
H	-0.35240240	1.46771586	-2.19316954
C	-0.82356309	1.74578891	0.45058643
H	-1.81320086	2.03420726	0.16365102
H	-0.80140701	1.62590600	1.51361851

Cyclopentane-1,3-dione_S

6	-0.346808	0.573944	-0.371776
6	1.659525	1.974614	0.173284
6	0.405454	2.799748	0.378752
1	-0.600101	-0.272554	0.274224
1	2.128087	1.789389	1.148789
1	2.405468	2.470820	-0.451006
6	1.185027	0.654372	-0.405618
8	1.903936	-0.206187	-0.841110

C	-4.98753227	1.70917638	2.19942351
H	-5.31977846	2.55143959	1.62924358
H	-5.83568566	1.17408683	2.57253877
H	-4.38991546	2.04733509	3.02003477

Alpha-keto acid

S	-0.26481602	1.55809996	0.14031850
O	1.08494196	2.02776652	-0.20215910
O	-1.09807955	2.70417831	0.53006260
N	-0.95996134	0.78311048	-1.21624491
H	-0.39296111	0.00325314	-1.48144911
C	-0.16679971	0.40780142	1.49516390
H	0.26817426	0.89273635	2.34400767
H	-1.14954401	0.06584223	1.74451817
H	0.43989053	-0.42664594	1.21139540
C	-1.04090739	1.73307613	-2.33513409
C	0.02450369	1.84381883	-3.23876966
C	-2.18348704	2.52796736	-2.49817287
C	-0.05266629	2.74945031	-4.30544598
H	0.89688817	1.23690098	-3.11428654
C	-2.26065767	3.43359773	-3.56485009
H	-2.99695063	2.44341528	-1.80822510
C	-1.19524741	3.54433900	-4.46848680
H	0.76079841	2.83400430	-4.99539221
H	-3.13304223	4.04051543	-3.68933332
H	-1.25416885	4.23580797	-5.28291847

Aryl sulfonamides

C	-1.31616353	-1.44458610	-0.03206510
C	0.08478254	-1.47872710	-0.04238303
C	0.81498164	-0.28271638	-0.02536564
C	0.14423467	0.94743534	0.00196988
C	-1.25671140	0.98157634	0.01228755
C	-1.98691050	-0.21443438	-0.00472990
H	-1.87368674	-2.35776686	-0.04505657
H	0.59691274	-2.41797524	-0.06325387
H	1.88463504	-0.30878376	-0.03324500
H	-1.76884160	1.92082448	0.03315818
B	1.00396674	2.35561121	0.02200657
O	1.39595424	3.03736253	-1.30206327
H	1.44756688	3.98684604	-1.17008900
O	1.41439584	2.98815738	1.36477733
H	1.46442121	3.94187599	1.26721569
C	-3.52641168	-0.17691680	0.00660828
H	-3.85807697	0.69571103	0.52950293
H	-3.88993756	-0.14961836	-0.99937568
H	-3.90087392	-1.05077565	0.49757542

Boronic acid 1

B	1.00396677	2.35561125	0.02200695
O	1.39595787	3.03736068	-1.30206280
H	1.44757021	3.98684437	-1.17008973
O	1.41439228	2.98815929	1.36477792
H	1.46441796	3.94187776	1.26721507
C	0.14423469	0.94743538	0.00196992
H	-0.90262035	1.16849310	0.01328462
H	0.38286269	0.39643131	-0.88366611
H	0.39493849	0.36420102	0.86329755

Boronic acid 2

C	1.48060000	0.89740000	0.22510000
C	2.77260000	1.07310000	-0.59340000
C	3.27000000	2.43300000	-0.07090000
C	2.01600000	3.20640000	0.37760000
C	3.76810000	-0.08200000	-0.37870000
H	4.66590000	0.11430000	-0.92690000
H	3.99760000	-0.16620000	0.66300000
H	3.33270000	-0.99670000	-0.72310000
O	4.46240000	2.83150000	-0.01770000
O	1.97980000	4.36300000	0.87210000

O	0.77991352	2.26370164	0.01832269
O	-1.57229649	2.49314195	0.43513160
C	-0.36994055	0.36519218	1.48309125
H	-1.29573533	-0.16260085	1.57920864
H	0.41688355	-0.32965387	1.27573455
H	-0.15782147	0.88234782	2.39548127
N	-0.83062073	0.71231774	-1.30778274
H	-0.09527110	0.06292891	-1.50157405
C	-0.93111433	1.68153330	-2.40842902
O	-1.33257635	2.85231887	-2.18114079
C	-0.54509367	1.26412242	-3.83963698
H	0.21479816	0.51191274	-3.79907838
H	-1.40586820	0.87481746	-4.34204006
C	-0.01387735	2.48963916	-4.60613417
H	0.25433181	2.19961991	-5.60054489
H	-0.77376918	3.24184884	-4.64669276
H	0.84689718	2.87894412	-4.10373108

Acyl sulfonamides 1

S	-0.35420000	1.55080000	0.15080000
O	0.98720000	2.11060000	-0.06580000
O	-1.28670000	2.63300000	0.49600000
N	-0.88540000	0.77450000	-1.27720000
H	-0.25090000	0.03820000	-1.51210000
C	-0.93300000	1.74960000	-2.37630000
O	-1.16950000	2.96130000	-2.13260000
C	-0.69330000	1.28820000	-3.82590000
H	-0.03260000	0.44650000	-3.82770000
H	-1.62610000	1.01030000	-4.27050000
C	-0.06220000	2.43790000	-4.63290000
H	0.10430000	2.11730000	-5.64010000
H	-0.72290000	3.27960000	-4.63110000
H	0.87050000	2.71580000	-4.18820000
C	-0.29670000	0.37020000	1.48170000
C	-1.46290000	-0.30030000	1.87450000
C	0.91490000	0.11110000	2.13660000
C	-1.41760000	-1.22980000	2.92230000
H	-2.38790000	-0.10250000	1.37450000
C	0.96020000	-0.81840000	3.18440000
H	1.80530000	0.62300000	1.83670000
C	-0.20600000	-1.48890000	3.57730000
H	-2.30800000	-1.74170000	3.22230000
H	1.88520000	-1.01620000	3.68450000
H	-0.17140000	-2.19860000	4.37730000

Acyl sulfonamides 2

S	-0.48401037	1.53783908	0.15044829
O	0.79993116	2.24045306	0.01796474
O	-1.54768123	2.51082565	0.43615026
C	-0.38212183	0.36184119	1.48275744
H	-1.31694589	-0.14972451	1.57921729
H	0.39232551	-0.34657989	1.27474090
H	-0.16049750	0.87490789	2.39519660
N	-0.83819505	0.71789137	-1.30774887
H	-0.11441248	0.05581559	-1.50215686
C	-0.92233896	1.68908064	-2.40802666
O	-1.30315489	2.86662757	-2.18013849
C	-0.54445624	1.26546309	-3.83958274
H	-1.11607227	1.83462962	-4.54257959
H	0.49830198	1.44327820	-4.00063264
H	-0.75304356	0.22414977	-3.97018860

Acyl sulfonamides p1

C	-4.83290080	-0.35054972	0.51169797
O	-4.05404185	-1.21300200	-0.32164157
H	-4.59022205	-1.51639665	-1.05789050
O	-6.07733682	-0.51572306	0.59926131
C	-4.14876078	0.78038024	1.30198263
O	-2.90432452	0.94555246	1.21442068

H	0.73955047	0.41475032	-4.53636824
H	-0.25563501	1.84628941	-4.65198982
H	1.24942433	1.89779660	-3.76586903

Trifluoromethyl sulfonamide

C	-2.35935299	1.39519331	0.50219429
O	-1.79918647	2.12752628	1.35862436
C	-3.76437807	1.75347674	-0.01659034
H	-3.89451295	2.81519217	0.01034020
H	-3.87224838	1.40633166	-1.02294728
H	-4.50259162	1.28784358	0.60238181
N	-1.67255121	0.19772072	-0.00304101
H	-1.96384290	0.01788233	-0.94261927
C	-0.21935679	0.41746900	0.02588406
N	0.91413485	0.58887272	0.04844556

Acyl cyanamide

C	4.78980000	-0.87580000	-0.02300000
C	3.35400000	-0.41810000	-0.04040000
C	3.02540000	0.80610000	-0.59200000
C	1.70840000	1.22590000	-0.60780000
C	0.72000000	0.42130000	-0.07250000
C	1.04850000	-0.80330000	0.47830000
C	2.36500000	-1.22500000	0.49030000
S	-0.95850000	0.95690000	-0.09160000
O	-1.14060000	1.24730000	1.28740000
N	-1.72660000	-0.50750000	-0.18100000
C	-3.07270000	-0.56110000	-0.21920000
O	-3.72160000	0.46310000	-0.18800000
C	-3.77170000	-1.89370000	-0.30050000
H	5.26760000	-0.53820000	0.89660000
H	4.82480000	-1.96410000	-0.07250000
H	5.31530000	-0.45600000	-0.88080000
H	3.79760000	1.43470000	-1.01020000
H	1.45180000	2.18260000	-1.03810000
H	0.27640000	-1.43180000	0.89690000
H	2.62160000	-2.18180000	0.92060000
H	-1.20770000	-1.32660000	-0.20590000
H	-3.96740000	-2.26340000	0.70600000
H	-4.71480000	-1.77730000	-0.83460000
H	-3.13860000	-2.60430000	-0.83190000

Acyl sulfinamide_R

C	-3.52376936	-0.76836031	-0.33691073
O	-2.48107854	-0.78460403	0.36743475
C	-4.78349264	-1.51805394	0.13495442
H	-5.38803956	-0.86261085	0.72640804
H	-5.34212855	-1.84764872	-0.71604101
H	-4.49557211	-2.36479328	0.72235058
N	-3.53932555	-0.03376857	-1.61010867
H	-3.25052329	-0.64202946	-2.34944076
S	-2.46798308	1.29557334	-1.51432027
O	-2.48353503	2.02996519	-2.78717175
C	-0.81987972	0.70712271	-1.18892638
C	-0.14557283	1.11662021	-0.03067599
C	-0.19662929	-0.16566396	-2.09099306
C	1.15198402	0.65333004	0.22550835
H	-0.62143847	1.78301198	0.65807208
C	1.10092790	-0.62895339	-1.83480919
H	-0.71147698	-0.47832315	-2.97534373
C	1.77523435	-0.21945682	-0.67655821
H	1.66683108	0.96598795	1.10985982
H	1.57679320	-1.29534587	-2.52355680
C	3.20112094	-0.72856701	-0.39503687
H	3.15085567	-1.63543449	0.17061743
H	3.70323376	-0.91352895	-1.32162837
H	3.73998679	0.00952999	0.16150280

Acyl sulfinamide_S

S	-0.49162668	1.53880012	0.15033588

C	-0.56808472	2.28625943	1.09745000
H	-1.61205478	2.34306045	1.32504649
H	-0.03288369	2.99018105	1.69990681
H	-0.41567262	2.51353777	0.06303465
N	-0.17286832	-1.39360537	0.09372575
H	0.81056837	-1.23894202	-0.00078107
O	-0.68734843	-1.78457960	-1.10295690
H	-0.26114879	-2.59748493	-1.38427309
H	-0.20760929	0.63699841	2.42599389

Hydroxamic acid 2

S	-0.33085764	0.88974715	0.22055712
O	0.91606120	1.60905697	-0.07526539
O	-1.47883548	1.70907857	-0.19244054
C	-0.49429947	1.57535651	4.14632158
H	-0.48174855	2.50215177	4.68091772
H	-1.40216770	1.05163475	4.36170678
H	0.34153088	0.97881010	4.44702075
N	-0.42592558	0.58055210	1.89968205
H	-1.27440056	0.09109251	2.10097663
C	-0.40868273	1.85381287	2.63412721
H	-1.24451308	2.45035929	2.33342803
H	0.49918550	2.37753464	2.41874201
C	-0.35173674	-0.65202439	-0.66877103
H	-0.29224978	-0.45855147	-1.71945154
H	0.48409362	-1.24857081	-0.36807186
H	-1.25960497	-1.17574616	-0.45338583

Sulfonamides (with methyl)

S	-0.40860071	1.59025214	0.16501260
O	0.86533310	2.31740764	0.25479951
O	-1.52330815	2.53095148	0.34455711
C	-0.47212903	0.34549330	1.43581533
H	-1.39966658	-0.18394082	1.37044245
H	0.33947750	-0.33941980	1.30509090
H	-0.39438640	0.81158662	2.39582216
N	-0.53284361	0.84537404	-1.36920391
H	-0.49715353	1.54467676	-2.08313804
H	0.22566717	0.20526834	-1.49137628

Sulfonamides

S	-0.50030000	1.54060000	0.14630000
O	0.76080000	2.28260000	0.00890000
O	-1.59270000	2.48030000	0.43470000
C	-0.35750000	0.36960000	1.47930000
H	-1.27560000	-0.17070000	1.57930000
H	0.43790000	-0.31460000	1.26930000
H	-0.14890000	0.89020000	2.39050000
N	-0.83370000	0.70860000	-1.31000000
H	-0.09030000	0.06910000	-1.50630000
C	-0.95160000	1.67560000	-2.41090000
O	-1.36800000	2.84100000	-2.18270000
N	-0.58300000	1.28130000	-3.77820000
H	-1.38430000	0.90620000	-4.24440000
C	-0.09470000	2.45740000	-4.51250000
H	0.17350000	2.17040000	-5.50780000
H	-0.86490000	3.19920000	-4.55060000
H	0.76260000	2.85870000	-4.01370000

Sulfonylureas

C	-0.10354288	-0.46701866	-0.30001245
F	0.73105381	-1.16787593	0.49669795
F	-1.16785284	-1.23286164	-0.62130492
F	-0.52455404	0.63827152	0.35082025
S	0.75444934	0.00950719	-1.78499476
O	1.91304928	0.84319818	-1.43523815
O	1.21275897	-1.19370352	-2.49348642
N	-0.30270646	0.89725974	-2.79416127
H	-0.61456658	1.71599321	-2.31206297
C	0.40586014	1.29079513	-4.02052306

C	-2.69285906	0.01828029	0.25983164
H	1.16603531	-0.10646691	0.52941509
H	-0.04228183	1.98034937	1.05943919
H	-2.50959908	2.04285854	0.94730829
H	-3.76022602	0.05083493	0.19224489

4-hydroxyquinoline-2-one

C	0.07195684	0.81061399	-0.10422395
C	1.02021398	0.92503822	-1.05244693
C	1.62010860	2.29851681	-0.89359102
C	0.97575732	2.87989513	0.13510816
O	-0.15182456	2.07489387	0.58157217
H	1.30144919	0.18262134	-1.76981987
H	2.41100675	2.72299012	-1.47600197
C	1.38226106	4.22609190	0.76288914
H	0.51328204	4.71608845	1.14977265
H	2.07787101	4.05354445	1.55740420
H	1.83807103	4.84398731	0.01767607
C	-0.67960578	-0.49462050	0.21689369
H	-0.75394234	-1.09051315	-0.66870556
H	-0.14589148	-1.03783340	0.96853775
H	-1.66117313	-0.26239867	0.57396310

5-methylfuran

C	1.36444203	0.11977486	0.29530003
C	0.72757514	2.16486401	-0.18601290
N	-0.34749615	1.44949309	0.00538627
C	0.74371026	3.70238135	-0.27192045
H	0.61436961	4.00407867	-1.29032593
H	-0.05198209	4.10115936	0.32200698
H	1.67995400	4.07018104	0.09286858
N	1.90809408	1.32634698	-0.28750131
H	2.70361835	1.69447261	0.19377269
O	2.04193460	-0.77392749	0.86615072
O	-0.06266274	0.09846787	0.14262391

5-oxo-1,2,4-oxadiazole

C	1.48808176	0.04404584	0.31479414
C	0.68131683	2.17249611	-0.19102660
N	-0.47091366	1.54921799	-0.04171451
C	0.76709400	3.70871750	-0.25634200
H	0.68222876	4.02772600	-1.27414919
H	-0.02824098	4.13511381	0.31857404
H	1.70689266	4.03068730	0.14116766
N	1.90124522	1.31362174	-0.28003412
H	2.67488833	1.72225183	0.20421559
O	2.25401296	-0.78409444	0.87256080
S	-0.24690587	-0.13078368	0.14525419

5-oxo-1,2,4-thiadiazole

C	-1.39773494	1.04614272	0.66335923
C	-0.32445991	2.87430856	-0.00959310
N	-1.05228158	0.82973425	-0.58191862
N	-0.17861611	1.84515537	-1.03900051
S	-0.08214112	4.40890504	-0.20935355
C	-2.39005661	0.16409969	1.44367826
H	-2.14397154	0.18555572	2.48477469
H	-3.38365475	0.53590313	1.30430492
H	-2.33201378	-0.84200786	1.08412490
C	1.20927614	1.36726010	-1.11819161
H	1.27445612	0.57883514	-1.83863428
H	1.84785635	2.17357592	-1.41309855
H	1.51575044	1.00151349	-0.16048449
N	-0.74833414	2.23714015	1.22202451
H	0.03088454	2.00829735	1.80550478

BACE1 carboxylic bioisosteres 2

C	-0.82548652	-0.14884409	0.52449116
O	-2.01359964	0.08887486	0.18471554
C	-0.05519718	0.86427674	1.39157854
H	0.98877287	0.80747573	1.16398205

O	2.98078266	-1.02910743	0.00197295
H	3.30099257	-1.02869891	-0.90304940
C	-2.79201715	-1.02842593	0.00063468
H	-3.14869733	-0.15498097	0.50538526
H	-3.14845066	-1.02788707	-1.00825325
H	-3.14890341	-1.90228343	0.50452481

2,6-difluorophenols1

C	-0.55182167	-2.24209999	-0.00064151
C	0.84952657	-2.24200729	0.01140341
C	1.55017823	-1.02833159	0.01069843
C	0.84948165	0.18525142	-0.00204978
C	-0.55186659	0.18515874	-0.01409370
C	-1.25251825	-1.02851697	-0.01338957
H	-1.08686400	1.11175631	-0.02382577
F	1.52443507	1.35441242	-0.00272684
F	1.52452328	-3.41107899	0.02368320
O	2.98012542	-1.02823702	0.02298743
H	3.30789195	-1.03323199	-0.87931194
C	-1.32176854	-3.57580956	0.00013116
H	-1.50876041	-3.87910894	1.00906303
H	-2.25233854	-3.45072512	-0.51299004
H	-0.73916975	-4.32426296	-0.49514267
H	-2.32247873	-1.02858774	-0.02258547

2,6-difluorophenols 2

C	-0.53875981	-0.48547099	0.27242879
C	0.59505715	1.14190618	-0.59119661
O	-0.76060500	0.72915016	-0.46827441
N	0.65618337	-0.58042776	0.79107950
N	1.40401976	0.55813006	0.47083809
H	1.48511459	1.17906873	1.25049109
S	1.14454756	2.07832046	-1.72026594
C	-1.60909569	-1.58206334	0.42561601
H	-1.46790441	-2.09074554	1.35631866
H	-2.58164758	-1.13632106	0.40673100
H	-1.52141001	-2.28104148	-0.37976638

2-thioxo-1,3,4-oxadiazole

C	-0.49020000	1.36530000	-0.10840000
C	0.84150000	1.28370000	-0.06080000
C	1.32080000	2.70790000	-0.01580000
O	-0.90380000	2.74770000	-0.27980000
H	1.43870000	0.39590000	-0.04540000
N	0.27040000	3.46210000	-0.04200000
O	2.68020000	3.14550000	0.05840000
H	2.94140000	3.22380000	0.97890000
C	-1.45030000	0.16660000	0.00520000
H	-1.70020000	0.00600000	1.03310000
H	-2.34150000	0.36950000	-0.55110000
H	-0.97620000	-0.70860000	-0.38750000

3-hydroxyisoxazole

C	-2.09918701	-3.39438348	-1.14772426
C	-0.56933739	-3.43359301	-1.00704568
C	-2.78300925	-2.34330389	-0.65933701
O	-4.21237424	-2.32567136	-0.69812877
H	-4.55919912	-2.70849970	0.11104054
C	-2.84308173	-4.54285785	-1.85428443
H	-2.91746713	-4.32949791	-2.90015457
H	-2.30406964	-5.45648597	-1.71410731
H	-3.82457033	-4.64055562	-1.43951309
O	0.08939186	-4.29696466	-1.64284103
N	0.13797032	-2.48475545	-0.12578769
H	1.02852923	-2.27016120	-0.52685393
C	-2.00470508	-1.15245643	-0.08630225
C	-0.60973733	-1.22398533	0.07690480
C	0.09865071	-0.07613189	0.46107839
C	-0.58677152	1.10780355	0.76432095
C	-1.98412020	1.14827328	0.68560394

C	1.59310000	1.56100000	0.52770000
C	0.76580000	0.73660000	-0.46070000
H	1.12470000	0.88830000	-1.47870000
C	0.80860000	-0.73350000	-0.09460000
O	1.78640000	-1.44840000	-0.04530000
N	-0.46810000	-1.07960000	0.16560000
C	-1.29510000	-0.03830000	0.01400000
O	-2.49520000	-0.09760000	0.19120000
O	-0.64860000	1.08210000	-0.35870000
H	2.63550000	1.24620000	0.48020000
H	1.52050000	2.61750000	0.26970000
H	1.21260000	1.40660000	1.53740000
H	-0.75100000	-1.96940000	0.42870000

2,4-oxazolidinedione_R

C	0.96370000	-0.02640000	0.58310000
C	3.01040000	0.62940000	0.03450000
C	0.87120000	1.22820000	-0.28820000
O	-0.19010000	1.83920000	-0.57760000
C	-0.17880000	-1.02330000	0.31390000
H	-1.11720000	-0.55770000	0.53210000
H	-0.15840000	-1.32010000	-0.71390000
H	-0.05470000	-1.88460000	0.93640000
N	2.20790000	1.61000000	-0.71240000
O	4.19930000	0.81640000	0.40190000
H	0.93240000	0.26360000	1.61260000
H	2.41890000	2.56680000	-0.51260000
O	2.25720000	-0.58110000	0.28740000

2,4-oxazolidinedione_S

C	2.03460000	-1.19660000	-0.67960000
C	1.11670000	-0.60740000	0.39330000
H	1.52270000	-0.77100000	1.39150000
C	0.84630000	0.86900000	0.14070000
O	1.72970000	1.69930000	0.14430000
N	-0.41840000	1.18350000	-0.08340000
C	-1.41510000	0.27900000	-0.09270000
O	-2.59970000	0.47070000	-0.28770000
S	-0.59070000	-1.27050000	0.24950000
H	3.01030000	-0.71330000	-0.62840000
H	2.15020000	-2.26720000	-0.51100000
H	1.59740000	-1.02890000	-1.66390000
H	-0.63910000	2.11340000	-0.24930000

2,4-thiazolidinedione_R

C	0.88919151	-0.07829097	0.62577049
C	3.19347817	0.77868791	0.01333413
C	0.88205332	1.17480993	-0.27984020
O	-0.17736427	1.76952074	-0.60774870
C	-0.26422447	-1.05135169	0.31855003
H	-1.19978335	-0.55797838	0.48045234
H	-0.20049997	-1.36943433	-0.70108845
H	-0.19378950	-1.90273001	0.96282783
N	2.23325144	1.63149767	-0.72106779
O	4.35774780	1.12426012	0.34291968
H	0.81278610	0.23299558	1.64663428
H	2.35713739	2.60671201	-0.53773949
S	2.47191583	-0.79829857	0.35409586

2,4-thiazolidinedione_S

C	-0.55146036	-2.24233830	0.00055474
C	0.84993959	-2.24250374	0.00088042
C	1.55078271	-1.02893862	0.00164137
C	0.85022587	0.18479195	0.00207736
C	-0.55117408	0.18495739	0.00175005
C	-1.25201720	-1.02860773	0.00099050
H	-1.08656963	-3.16892220	-0.00002621
H	-1.08606478	1.11166762	0.00208056
F	1.52536373	1.35384641	0.00281290
F	1.52480168	-3.41171757	0.00046119

C	-0.66232015	0.32834283	0.08677107
C	0.04496564	2.43953947	0.01183061
N	-1.07598819	1.65768683	0.51935935
H	-1.96471200	1.94265980	0.16024879
H	0.03439903	3.49898458	-0.13771671
N	0.60528448	0.32203745	-0.19498369
N	1.05477109	1.66380914	-0.24272929
C	-1.59644726	-0.89038477	-0.03031684
H	-2.38024773	-0.80985871	0.69361160
H	-2.01991206	-0.92214065	-1.01244164
H	-1.03821836	-1.78593322	0.14652624

1,2,4-triazole

C	1.82769072	-0.87716719	-1.09002261
C	2.52439391	0.34479191	-0.52491222
C	1.61072373	0.97254799	0.21314851
H	2.27459076	-1.60881353	-1.73023663
H	3.53654787	0.64735033	-0.69492038
N	0.59208661	-0.88308419	-0.67797220
N	0.41809359	0.10477447	0.33928624
O	-0.73782492	0.80620517	0.19283704
H	-0.74506040	1.23545133	-0.66582202
C	1.76618266	2.37623814	0.82722220
H	1.41243786	3.11016040	0.13359209
H	1.19608731	2.43540422	1.73076559
H	2.79803647	2.55844099	1.04397049

1-hydroxypyrazole

C	-1.63310000	1.22490000	0.02230000
C	-0.88790000	-0.00930000	0.53430000
H	-0.87850000	-0.00190000	1.62430000
C	0.55000000	0.00990000	0.01180000
F	1.19540000	1.16550000	0.46460000
F	1.23140000	-1.11870000	0.47990000
F	0.53790000	0.00030000	-1.38720000
O	-1.54710000	-1.18980000	0.07170000
H	-1.64250000	1.21750000	-1.06760000
H	-2.65750000	1.21120000	0.39460000
H	-1.13030000	2.12530000	0.37520000
H	-1.53350000	-1.15700000	-0.89460000

2,2,2-triflouroethan-1-ol_R

C	-1.02851420	0.73563327	-0.14424596
F	-0.52407285	1.92870690	-0.52452775
F	-2.24199341	1.26317880	-0.41194713
F	-0.04432790	0.88415600	0.76779479
C	-1.72610688	0.07036526	1.05674652
O	-2.40917523	1.06611103	1.82278046
H	-1.77715705	1.71566820	2.13936933
C	-2.73996938	-0.97163268	0.54888521
H	-2.22886230	-1.71670120	-0.02430102
H	-3.22466040	-1.43386435	1.38334102
H	-3.47082238	-0.48831808	-0.06524905
H	-0.99525388	-0.41294933	1.67088078

2,2,2-triflouroethan-1-ol_S

C	-1.03704232	0.73493343	-0.15504991
F	-0.53044547	1.93358963	-0.51433104
F	-2.25069002	1.27527401	-0.39503180
F	-0.04051637	0.85694762	0.74747354
C	-1.72326019	0.04615542	1.03922389
O	-2.39068805	1.02784096	1.83655290
H	-1.75089359	1.66640415	2.15981769
C	-2.74959713	-0.97820635	0.52065329
H	-2.25019307	-1.71275427	-0.07594954
H	-3.22638487	-1.45677288	1.35044093
H	-3.48531767	-0.47682377	-0.07283705
O	-0.74000750	-0.62391662	1.83239322
H	-1.16777968	-1.05328472	2.57687559

2,2,2-triflouromethayl-ketones (hydrated)

H	0.77900000	0.79080000	-0.61300000
H	3.58240000	0.73560000	-2.84550000
H	2.91920000	1.54480000	-1.48630000
O	2.18700000	-1.54500000	-2.56350000
H	3.08600000	-1.81000000	-2.77130000
C	2.92150000	-1.07650000	-0.35890000
H	3.91190000	-1.38050000	-0.62660000
H	2.97210000	-0.33180000	0.40780000
C	2.13330000	-2.29380000	0.15910000
H	1.14290000	-1.98980000	0.42680000
H	2.08270000	-3.03850000	-0.60760000
H	2.62640000	-2.69900000	1.01790000
S	1.10340000	1.05440000	-2.97500000

Thietan-3-ol

C	-2.36550000	-1.78760000	0.95460000
C	-3.78870000	-1.27540000	0.66560000
H	-3.74310000	-0.25360000	0.35110000
H	-4.23060000	-1.86740000	-0.10840000
H	-4.38140000	-1.34910000	1.55340000
C	-1.28170000	-0.81200000	1.44990000
F	-0.07060000	-1.23210000	1.02650000
F	-1.29700000	-0.76970000	2.79910000
F	-1.52750000	0.42100000	0.95810000
O	-2.08800000	-3.00340000	0.78600000

Trifluoromethyl ketone
